# Fabrication of a Maxillary Prosthesis for a Flap-Reconstructed Maxillary Defect Using a 3D-Printed Occlusal Record Base

**DOI:** 10.7759/cureus.110660

**Published:** 2026-06-11

**Authors:** Mariko Hattori, Manjin Zhang, Yuka Sumita, Noriyuki Wakabayashi

**Affiliations:** 1 Department of Advanced Prosthodontics, Institute of Science Tokyo, Tokyo, JPN; 2 Stomatological Hospital, School of Stomatology, Southern Medical University, Guangzhou, CHN; 3 Department of Partial and Complete Denture, The Nippon Dental University, School of Life Dentistry at Tokyo, Tokyo, JPN

**Keywords:** complete edentulous, digital dentistry, digital workflow, flap reconstruction, maxillectomy, maxillofacial prosthesis, obturator

## Abstract

The fabrication of maxillary prostheses in patients with flap-reconstructed maxillary defects continues to present both clinical and technical challenges. Conventional impression techniques can be hazardous and often require multiple adjustments. This report presents a simplified digital approach using 3D scan data of an existing, well-adjusted prosthesis. The previously relined and adjusted prosthesis was scanned with an intraoral scanner, and the 3D surface data were used to print an occlusal record base. After recording the jaw relation, the teeth were arranged, and a functional impression was made at the try-in stage. Finally, the definitive prosthesis was fabricated using heat-cured acrylic resin. The printed record base provided accurate adaptation and minimized the clinical risks associated with conventional impression making. Incorporating the functional impression during the try-in stage further improved the fit and reduced the need for postdelivery adjustments. This technique offers a practical and simplified method for maxillary prosthesis fabrication. Reusing the morphology of a clinically adapted prosthesis eliminates complex digital processing and reduces clinical burden, making it especially useful in settings with limited access to advanced technical support.

## Introduction

The fabrication of maxillary prostheses for patients with complex maxillofacial defects remains a significant challenge, particularly during impression taking [1]. Even after completion, repeated adjustments are often required to accommodate the irregular morphology of the defect [2]. Multiple relinings or extensions of the denture base may also be necessary until a functional and stable form is achieved [2]. In such cases, it is reasonable to consider using the morphological information of a clinically adapted prosthesis to fabricate a new one [3].

Conventional impression making for maxillofacial prostheses carries inherent risks, including the entrapment of impression material within the defect or accidental ingestion and aspiration. These risks are particularly concerning for patients with surgically created or congenital defects, who often present with compromised anatomy. The incorporation of digital technology offers a promising alternative with the potential to reduce such risks [3]. In addition, limitation of mouth opening, a common postoperative complication, can significantly restrict clinical access during conventional impression making. Under such iatrogenic constraints, digital impression technology provides a viable alternative by reducing the need for maximal interarch opening [4].

Digital techniques have recently been applied not only to fixed but also to removable prostheses. Patzelt et al. demonstrated the use of intraoral scanners to capture mucosal surfaces for fabricating removable dentures on digital models [5]. Elbashti et al. confirmed the feasibility of intraoral scanning in model studies of maxillectomy patients [6,7], while Zhang et al. reported its limitations in capturing intraoral data in vivo [8]. Consequently, combining intraoral scans with other data sources, such as CT, has been proposed. Murat and Batak introduced the use of CT data for obturator fabrication [9], and Ding et al. described a digital workflow supplemented with thermoplastic wax impressions [10]. However, these protocols are technically demanding, requiring advanced skills in 3D data extraction, superimposition, and design.

As a simpler alternative, Elbashti et al. suggested scanning an existing denture rather than the defect directly [11,12]. This approach reduces the need for advanced data manipulation, making digital workflows more accessible to clinicians and dental technicians.

This report introduces a straightforward method for refabricating a maxillary prosthesis using 3D scan data of an existing obturator. This technique minimizes the clinical burden and technical complexity while ensuring accurate reproduction of defect morphology.

## Technical report

**Figure 1:**
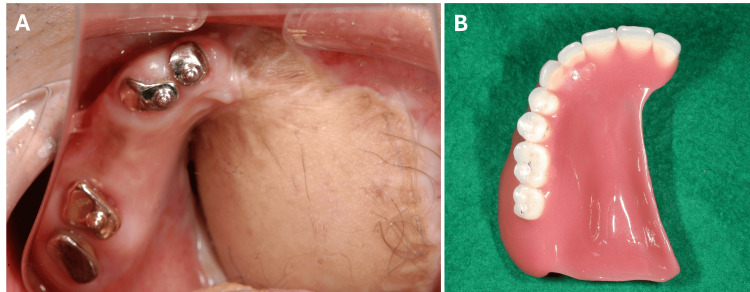
Patient’s intraoral view and the existing prosthesis (A) Intraoral view of the patient with a maxillary defect, including perforation and flap. (B) Occlusal view of the existing prosthesis.

The patient was a 65-year-old man with a left maxillary defect following resection of maxillary sinus carcinoma, which was reconstructed with an abdominal flap (Figure 1). He had been wearing a maxillary prosthesis retained by the defect undercut, which had been repeatedly relined and adjusted with auto-polymerizing acrylic resins (Tokuyama Rebase II; Tokuyama Dental, Tokyo, Japan and Unifast III; GC, Tokyo, Japan).

**Figure 2:**
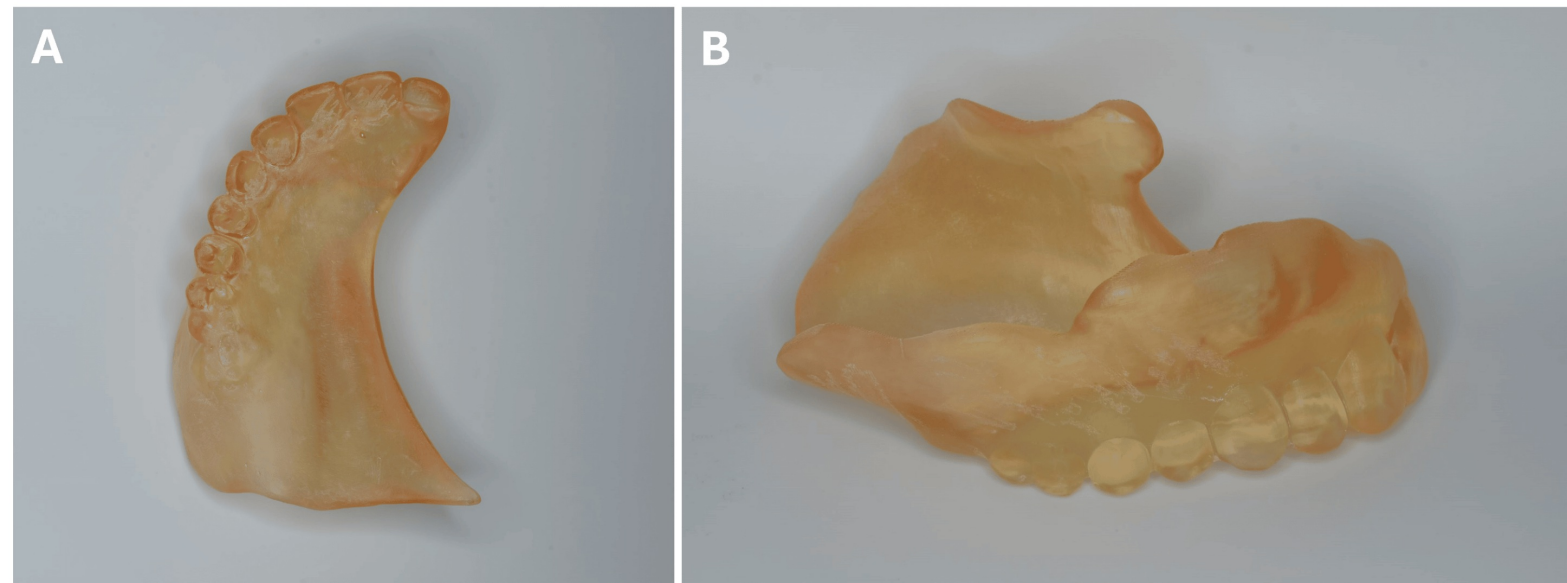
3D-printed replica of the existing prosthesis (A) Occlusal view. (B) Lateral view.

The existing prosthesis was adjusted intraorally to achieve optimal fit. Both the intaglio and polished surfaces of the adjusted prosthesis were scanned with an intraoral scanner (Trophy3DI; Yoshida, Tokyo, Japan) to obtain 3D data. The data were printed using a methacrylate ester-based UV-curable resin (VarseoWax Surgical Guide; Bego, Bremen, Germany) and a dental 3D printer (Varseo; Bego) (Figure 2).

**Figure 3:**
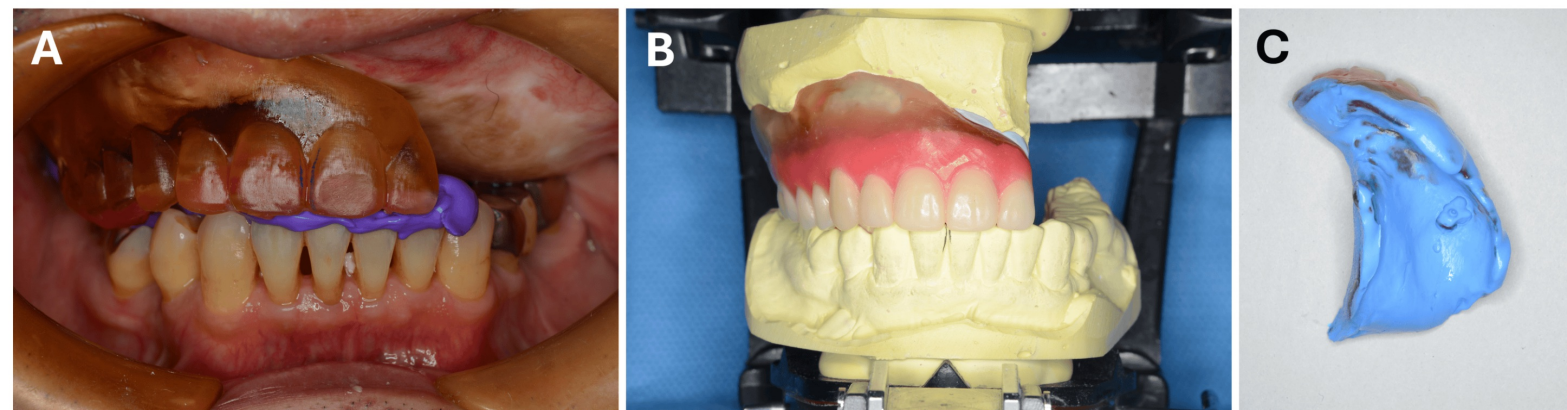
Steps using the printed prosthesis (A) Occlusal registration using the printed prosthesis as a record base. (B) Wax try-in prosthesis. (C) Intaglio surface of the functional seated impression.

The printed prosthesis was adjusted and used as an occlusal record base. The maxillomandibular relationship was recorded using silicone bite material (Correct Plus Bite; Pentron Japan, Tokyo, Japan) (Figure 3A).

**Figure 4:**
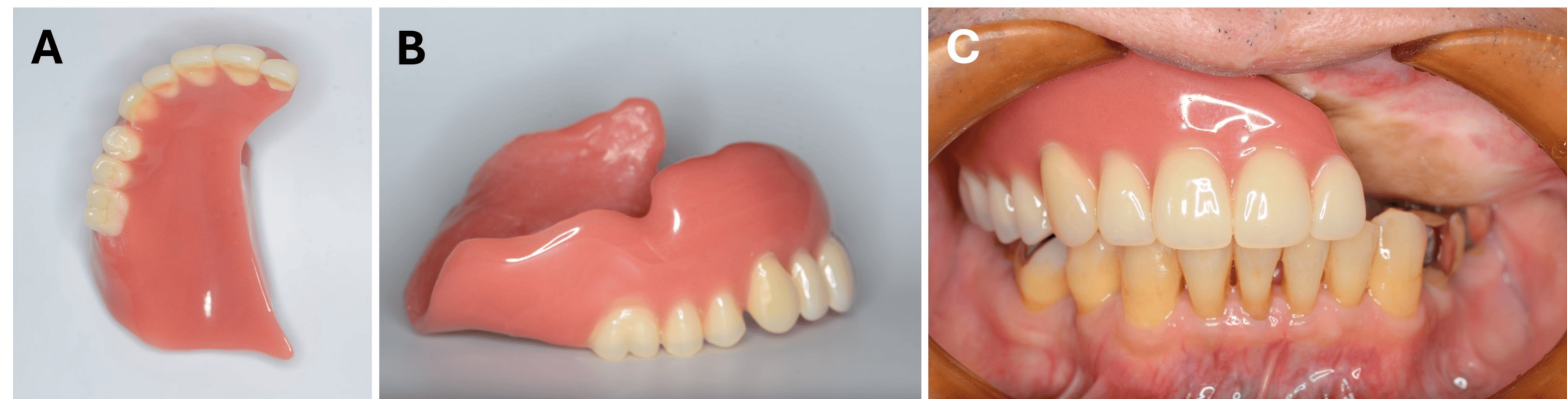
Completed prosthesis (A) Occlusal view. (B) Lateral view. (C) Delivered to the patient’s mouth.

The teeth segment of the printed prosthesis was removed, and artificial teeth (Endura Anterio and Endura Posterio; Shofu, Kyoto, Japan) were arranged in wax (paraffin wax; GC) to fabricate a wax try-in. The wax try-in was clinically evaluated and adjusted for fit, occlusion, and esthetics (Figure 3B). A functional impression was made on the intaglio surface during the try-in stage using silicone material (Examix Fine Regular; GC) (Figure 3C). The definitive prosthesis was fabricated with conventional heat-cured acrylic resin (Acron Dark Pink; GC), finished, polished, and delivered (Figure 4).

Minor intraoral adjustments and oral function tests [13] were performed, and the patient was scheduled for regular follow-up.

## Discussion

Oral function tests [13] revealed a moisture value of 25.4. The mean tongue pressure was 24.0 kPa, and the mean lip pressure was 4.8 kPa. The glucose concentration of the gummy jelly after chewing was 110.0 mg/dL. Oral diadochokinesis rates were /pa/ 3.0, /ta/ 2.4, and /ka/ 2.6 times/s. Maximum and average occlusal forces were 35.7 N and 25.9 N, respectively, with 100% on the right side. It is remarkable that the glucose concentration was above the threshold for hypofunction, although the other test results were below the threshold [13]. The decreases in lip pressure, tongue pressure, and occlusal force are likely due to the surgery. However, mastication met the criteria, indicating that the patient was able to compensate using the prosthesis to achieve sufficient mastication for nutrition. Although the overall results of the tests met the criteria for oral hypofunction, considering the post-maxillectomy condition, maintaining this level of function is clinically significant.

For the prosthetic rehabilitation of this patient, a combination of digital and conventional methods was used to fabricate the prosthesis. By utilizing the 3D data of the previous prosthesis as an occlusal record base, both the preliminary and final impression stages were omitted. The transparency of the printed material enabled direct visualization of mucosal adaptation, and the functional impression at the try-in stage provided enhanced accuracy. Consequently, the new prosthesis required minimal postdelivery adjustments and reduced the number of follow-up visits compared with the original prosthesis.

This approach is particularly valuable in challenging maxillary defect cases, where conventional impression techniques may be impractical or hazardous. In patients with severe undercuts or limited oral aperture, the morphology of an old, well-adapted prosthesis, often modified repeatedly in the oral cavity, can be successfully replicated [3]. The incorporation of digital scanning and 3D printing facilitated efficient fabrication while maintaining the functional form established over years of clinical use [3]. In this technique, an occlusal seating impression was performed using conventional impression material. Because the wax try-in prosthesis provided a stable fit, only a minimal amount of material was required, which allowed for a clear visual field during the impression procedure. This approach reduced the risk of material impaction, swallowing, and aspiration compared with conventional impression making using an individual tray, which typically requires larger amounts of material, often resulting in impaired visibility and increased difficulty for patients in controlling swallowing and aspiration.

Digital workflows generally require considerable knowledge and skill in handling 3D data to design and fabricate removable prostheses [9,10]. In contrast, the technique described in the present report involves simply printing the scanned surface data without modification, which may be the simplest way to utilize 3D data without the need for specialized software or expertise.

This approach allows clinicians to utilize the 3D morphological data from a well-fitting existing prosthesis, provided an intraoral scanner is available, even in cases where access to a dental technician with advanced digital skills is limited.

## Conclusions

The presented technique highlights the feasibility of fabricating a maxillary prosthesis by directly utilizing the 3D surface data of a previously adjusted prosthesis. This method eliminates the need for conventional impressions and complex digital design procedures, thereby reducing both technical demands and clinical risks. By reproducing the morphology of an old, well-fitting prosthesis, the approach enables efficient prosthesis fabrication with minimal postdelivery adjustments. It may represent a valuable option for clinicians managing maxillary defect cases, particularly when specialized digital expertise or technical resources are limited.
